# Pediatric precision oncology: “better three hours too soon than a minute too late”

**DOI:** 10.3389/fonc.2023.1279953

**Published:** 2023-10-30

**Authors:** Mark Marshall, Jennifer Ivanovich, Morgan Schmitt, Amy Helvie, Lisa Langsford, Jennifer Casterline, Michael Ferguson

**Affiliations:** ^1^ Division of Hematology/Oncology, Department of Pediatrics, Indiana University School of Medicine, Indianapolis, IN, United States; ^2^ Department of Medical and Molecular Genetics, Indiana University School of Medicine, Indianapolis, IN, United States; ^3^ The Medical Affairs Company, Kennesaw, GA, United States

**Keywords:** precision oncology, precision medicine, whole exome sequencing, pediatric cancer, targeted therapy

## Abstract

Precision oncology is defined as the selection of an effective treatment for a cancer patient based upon genomic profiling of the patient’s tumor to identify targetable alterations. The application of precision oncology toward pediatric cancer patients has moved forward more slowly than with adults but is gaining momentum. Clinical and pharmaceutical advances developed over the past decade for adult cancer indications have begun to move into pediatric oncology, expanding treatment options for young high-risk and refractory patients. As a result, the FDA has approved 23 targeted drugs for pediatric cancer indications, moving targeted drugs into the standard of care. Our precision oncology program is in a medium sized children’s hospital, lacking internal sequencing capabilities and bioinformatics. We have developed methods, medical and business partnerships to provide state-of-the-art tumor characterization and targeted treatment options for our patients. We present here a streamlined and practical protocol designed to enable any oncologist to implement precision oncology options for their patients.

## Introduction

Over 20 years ago the concept of precision medicine was introduced to the public through an insightful article first published in the Wall Street Journal and reprinted in The Oncologist (1). In essence, precision medicine is the selection of an effective treatment for a patient’s disease based upon an understanding of the patient’s individual genetics. Since that article, medical research has invested significant time and resources into attempting to realize the potential of personalized genome-based medicine. Oncology, more than any other single field of medicine, has tested this concept and found it to have significant benefit for the right patients. A targeted cancer drug is typically a small molecule or monoclonal antibody that interacts with a specific molecular target to achieve a desired effect either specifically within a cancer cell or with reduced toxicity toward normal cells. The current availability of over 163 FDA approved targeted drugs in 33 adult cancer indications (2, 3) has brought precision medicine for oncology out of the hypothetical stage into the tested and proven arena, at least for adult cancer patients.

## Pediatric precision oncology

The application of precision medicine in pediatric oncology (hereafter referred to as precision oncology) has moved forward more slowly than adult precision oncology with research efforts only recently gaining momentum. With the annual US cancer incidence in adults over 100-times greater than the incidence in children, research into cancer biology and the resultant industrial development of appropriately targeted drugs has been overwhelmingly directed toward adults (4). Outside of generalized risk factors, the genesis and genomic evolution of pediatric solid tumors remains less well understood than adult cancer origins. There is a growing consensus that pediatric cancer originates during tissue development, when multiple biochemical cell growth programs are active and easily altered by dysfunction (5). Significantly, children with birth defects are up to 12 times more likely to develop childhood cancer than children without birth defects (6). The types of cancers found in children as well as the corresponding genomic drivers also often deviate significantly from those found in adults, hindering the translation of genomic medicine from adult to pediatric oncology (7). Fortunately, some pediatric cancers do on occasion overlap the targetable cancer genome that is more frequently seen in adults and do benefit from precision guided therapeutic strategies (8, 9). This is particularly true in pediatric hematopoietic and central nervous system (CNS) malignancies. Furthermore, many pediatric cancer patients benefit diagnostically from the definitive molecular classification of their tumors now possible through genomic DNA and RNA sequencing (10–13). What may have once been considered histologically to be an undescribed soft tissue sarcoma or a histologically unclassified medulloblastoma can now be precisely identified as a gene-fusion specific sarcoma or one of four-distinct molecular subgroups of medulloblastoma, each with a preferred standard of care treatment and risk assessment (14, 15).

The progress leading up to the current state of the art of pediatric precision oncology is the result of many complementary studies focused on sequencing germline and tumor DNA, tumor transcriptome sequencing, methylome profiling and actionable target discovery. A recent comprehensive review on pediatric precision oncology cited 22 separately published DNA and RNA sequencing efforts in pediatric cancers (16). In large part, these publications document multi-center pediatric precision oncology studies conducted around the globe over the last decade involving over 3000 children, adolescents, and young adults. These studies focused primarily on genome sequencing and bioinformatic analysis with the goals of understanding the molecular basis for pediatric cancer and determining the prevalence of potentially actionable gene alterations in the pediatric cancer population with limited clinical translation.

Premier projects to understand the genomic landscape of pediatric cancer have been conducted by the Pediatric Cancer Genome Project (PCGP) (17), INFORM (INdividualized Therapy FOr Relapsed Malignancies in Childhood) (7), the Zero Childhood Cancer Program (ZCCP), MoleculAr Profiling for Pediatric and Young Adult Cancer Treatment Stratification (MAPPYACTS), a European prospective precision oncology trial (18), Pediatric MATCH (19) and others. These studies have shown many pediatric tumor types possess genome alterations which can be targeted by existing experimental drugs and approved adult oncology drugs off-label. It has been estimated that up to 50% of all pediatric cancers have at least one targetable mutation (17). Early work done by the ZCCP demonstrated that no less than 70% of children who received ZCCP-recommended personalized therapy showed a complete or partial response, or had their disease stabilized (20). FDA approval of 23 separate targeted drug entities for use in solid and hematopoietic pediatric cancers is the clearest sign that precision genomics has a valid role in modern pediatric oncology (2).

## Precision genomics in a small institutional setting

The guiding principle of precision oncology is that effective therapy may be determined independent of the cancer diagnosis if actionable genomic driver mutations are detected in the tumor. Through the generous publication of case studies, clinical trials and databases, a growing number of clinically tested, target-drug associations are now available for young patients with solid tumors with strongly oncogenic genomic biomarkers (21, 22). Most of these gene-drug associations are based on adult studies and will take years to fully validate in pediatric clinical trials. However, many safe and effective options are available today for use with children who have failed standard of care.

The promise of precision genomics in adult oncology prompted the creation of a precision genomics program at our hospital in 2016. The program was staffed with pediatric oncologists, nurse coordinators, a genetic counselor, a cancer molecular biologist, a pharmacologist, and an ethicist. The goal of this program was to provide molecular data to institutional oncologists for precise diagnoses and subtyping of cancers, identify targeted and chemotherapeutic drug sensitivities and resistance, predict sensitivity to immune checkpoint therapy and provide first pass germline genomic analysis to pediatric cancer patients who may have underlying cancer predisposition syndromes. In the early years of pediatric precision oncology, sequencing was limited to large in-house academic research centers. In the absence of in-house CLIA-certified genomic sequencing and bioinformatics at our institution, we developed partnerships with commercial CLIA-certified DNA/RNA sequencing and bioinformatic laboratories.

When we began our program, external CLIA-certified DNA sequencing providers were limited in number and scope. Most sequencing companies were focused on adult cancer genomic trends and not pediatric cancer. Initially we relied upon Paradigm Diagnostics, Inc. and Foundation Medicine to provide analysis of patient tumor samples. Both companies provided rapid turn-around and could sequence limited amounts of tumor tissue. However, the reliance of these companies upon adult-biased DNA and RNA sequencing panels meant that targetable findings were identified only in a minority of our patients. The common experience in the field has been that only a combination of somatic and germline DNA and RNA sequencing, supplemented with some protein analysis, will provide the most comprehensive biomarker information for pediatric cancer patients (20, 23, 24). Finding a single CLIA provider that provides somatic and germline DNA sequencing, methylome sequencing, RNA sequencing and limited protein analysis has so-far proven an impossible task. As of this writing, our precision oncology group relies upon Caris Life Sciences to provide somatic whole exome sequencing, RNA sequencing and disease-specific immunohistochemistry for most solid tumor patients (25). CNS tumors are often sent by our program to the Molecular Characterization Initiative (MCI), for sequencing of tumor and normal DNA, RNA sequencing using a fixed panel to identify fusions and microarray-based DNA methylation sequencing (26). When solid tumor sample size is limiting for standard sequencing, Foundation Medicine offers highly sensitive DNA and RNA panel-based sequencing which is highly effective for most pediatric CNS tumors (FoundationOne CDx) and sarcomas (FoundationOne Heme). When germline cancer predisposition is suspected, the patient is referred to our internal Medical and Molecular Genetics department.

To make tumor sequencing available to all patients, we have had to form relationships with multiple genomic sequencing companies to reduce or eliminate shared costs with our patients. Most sequencing companies will now work with insurance companies for approval of sequencing costs and have patient assistance programs to help defer the costs of sequencing. For instance, Caris Life Sciences, has a direct cost for DNA+RNA sequencing of $3100 per patient, but have kept average cost to the patient to just over $100. This direct cost is generously paid for by the Riley Children’s Foundation. Other companies we have worked with in the past have had similar costs, but over time increasing patient costs have propelled us to change providers. With the price dropping every year for CLIA certified somatic sequencing, this approach has been feasible with companies like Caris Life Sciences, Foundation Medicine, and Tempus, who attempt to keep the patient cost slow with assistance programs. Molecular characterization tumors through the Molecular Characterization Initiative is free of charge.

To aid with identifying targeted agents compatible with each patient’s genomic profile, each sequencing laboratory provides a clinical report with a list of pathogenic genome alterations and expressed gene fusions along with a list of FDA approved companion drugs and experimental agents that may be available through clinical trials. While useful to the pediatric oncologist in general, these reports are typically written with adult cancer patients in mind and fail to consider the age of the patient when it comes to FDA drug approvals and clinical trial age requirements. Furthermore, it is not unusual for significant errors to be present in these reports. For example, targetable gene variants are sometimes overlooked, and drug resistant gene mutations may be paired with inactive drugs. For this reason, we have found it advantageous to have a cancer genomic specialist review each report for errors and when possible, to reanalyze the genomic findings locally to identify pediatric-appropriate targeted therapies. Our in-house bioinformatics has been enabled through a software collaboration with LifeOmic, a healthcare technology company. Our DNA sequencing providers automatically upload the sequencing files for each patient to a secure Cloud-based server. LifeOmic Molecular Tumor Board software permits non-technologists to quickly examine patient data for additional genomic findings, unreported targeted drug opportunities and age/gene/cancer type specific clinical trials. Our criteria for pairing genomic biomarkers with targeted drugs is based upon the verified gene-drug lists available from the NCI (https://www.cancer.gov/about-cancer/treatment/drugs/childhood-cancer-fda-approved-drugs), the Memorial Sloan Kettering Cancer Center Precision Oncology Knowledge Base (OncoKB) (21) and the Jackson Laboratory Clinical Knowledgebase (JAX CKB) (22). Using these tools, our small institutional precision genomics program has provided over 500 tailored treatment recommendations to oncologists and their patients. More than 30% of these patients have received therapy guided by our genomic interpretation.

**Figure 1:**
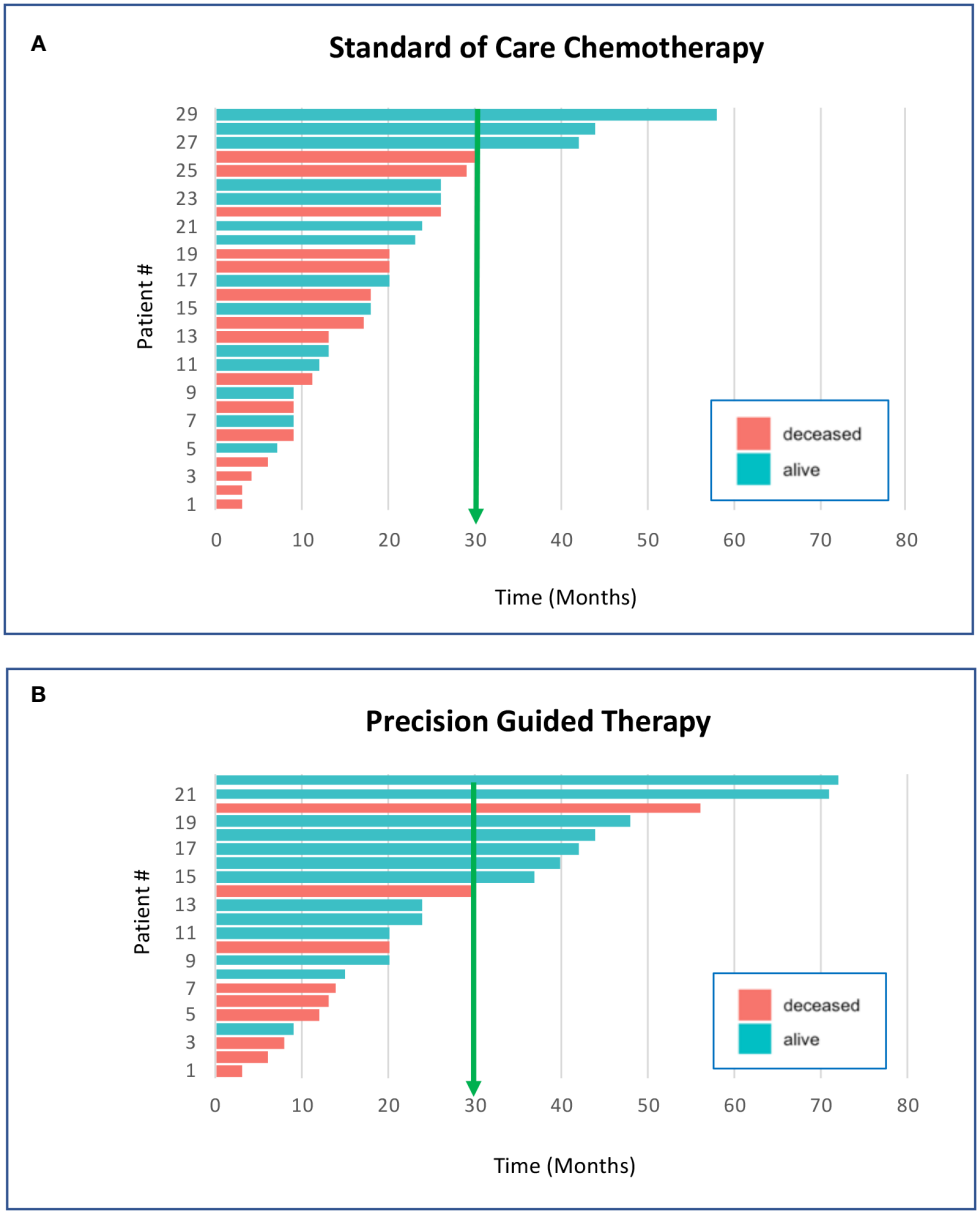
Survival of pediatric cancer patients is improved by precision genomics-guided therapy. These graphs compare the individual survival time in months for pediatric non-CNS solid cancer patients who have received standard of care chemotherapy **(A)** vs. precision genomics guided therapy **(B)**. The survival time for each patient was based upon entry into the Riley precision oncology program until the date-of-death, or of data analysis if still living. At the time of the analysis, there were 51 qualifying patients who had entered the program and received either chemotherapy or targeted agents as second-line therapy; 29 received second-line SOC chemotherapy and 22 received precision guided therapy (PGT) through the precision oncology program. Survival beyond 30 months was 10% for the SOC chemotherapy cohort vs 36% survival beyond 30 months for the PGT cohort. Average survival of the PGT cohort at the time of analysis was approximately 10 months longer than the SOC cohort (*p*=0.044 unpaired Student’s T-test; significant).

Because of the general effectiveness of cytotoxic chemotherapy, ethical considerations dictate that precision oncology-directed therapies should begin with disease progression or relapse following first line therapy. Our overwhelming clinical experience with precision genomics has been that when highly validated actionable genomic biomarkers are present in the patient tumor sample, appropriately targeted drugs used as single agents will succeed when first line standard of care (SOC) has failed. Our patients receiving genomic guided therapies following relapse typically demonstrate improved survival benefit when compared to relapsed patients receiving 2^nd^ line SOC chemotherapy (**Figure 1**). The average survival time of our initial precision guided therapy cohort at the time of analysis was 29 months verses 19 months for the SOC chemotherapy cohort (*p=0.044* unpaired Student’s T-test). 36% of the precision guided therapy cohort survived beyond 30 months versus only 10% for the SOC chemotherapy cohort. Similar results are accruing for our CNS cancer patients; however, more time is needed before we can accurately determine statistical significance (data not shown). We still do not know what targeted agent is the correct match or best available for molecular alterations when paired with chemotherapy. This needs to be investigated in small basket trials like the now open NIH ComboMATCH trial that can pair agents together to better inhibit a target or rescue pathways in tumor cells. These efforts are also aided by large scale data collection of molecular findings, therapy, and outcomes in pediatric patients. In addition, a number of pediatric cancer genomic data sharing efforts are underway, such as the Precision Oncology Alliance (Caris Lifesciences), the Children’s Oncology Group, the Beat Childhood Cancer group and the Pediatric Cancer Data Commons. With time, correlation of patient molecular markers, targeted therapeutics, chemotherapy and outcomes is anticipated to advance the treatment of many pediatric cancer types.

With the development of a novel pediatric precision genomics program, we have naturally experienced obstacles which have slowed down acceptance of genomics as a valid consideration for pediatric solid tumor treatment. In the earliest program days, a key challenge was convincing institutional oncologists that precision oncology had demonstrated value in patient care. Providing educational opportunities to the staff oncologists and fellows has succeeded in bringing precision oncology into our mainstream weekly Tumor Board discussions. Other challenges to widespread acceptance of using targeted drugs in pediatric settings include a paucity of clinical data to guide safe combination of targeted agents with traditional chemotherapy, a lack of insurance approval of targeted agents due to limited clinical data in pediatric cancer, and an unstable marketplace of CLIA-certified sequencing providers with shifting products and patient costs. Important medical reasons for not following program recommendations were that high risk patients were already in remission from 1^st^ line therapy, radiation and/or surgery or that relapsed patients were already receiving second line chemotherapy when genomic analysis was performed.

## A pediatric oncology care team user guide for applying precision genomics

With eight years of clinical experience in pediatric precision oncology, our institutional precision genomics program stands behind the belief that ‘now’ is the time to include genomic methodologies broadly across pediatric oncology programs and practices. We have worked diligently with pediatric oncologists, drug and insurance companies and families to develop an effective in-house precision genomics program that could be applied at most smaller centers and oncology practices.

**Figure 2:**
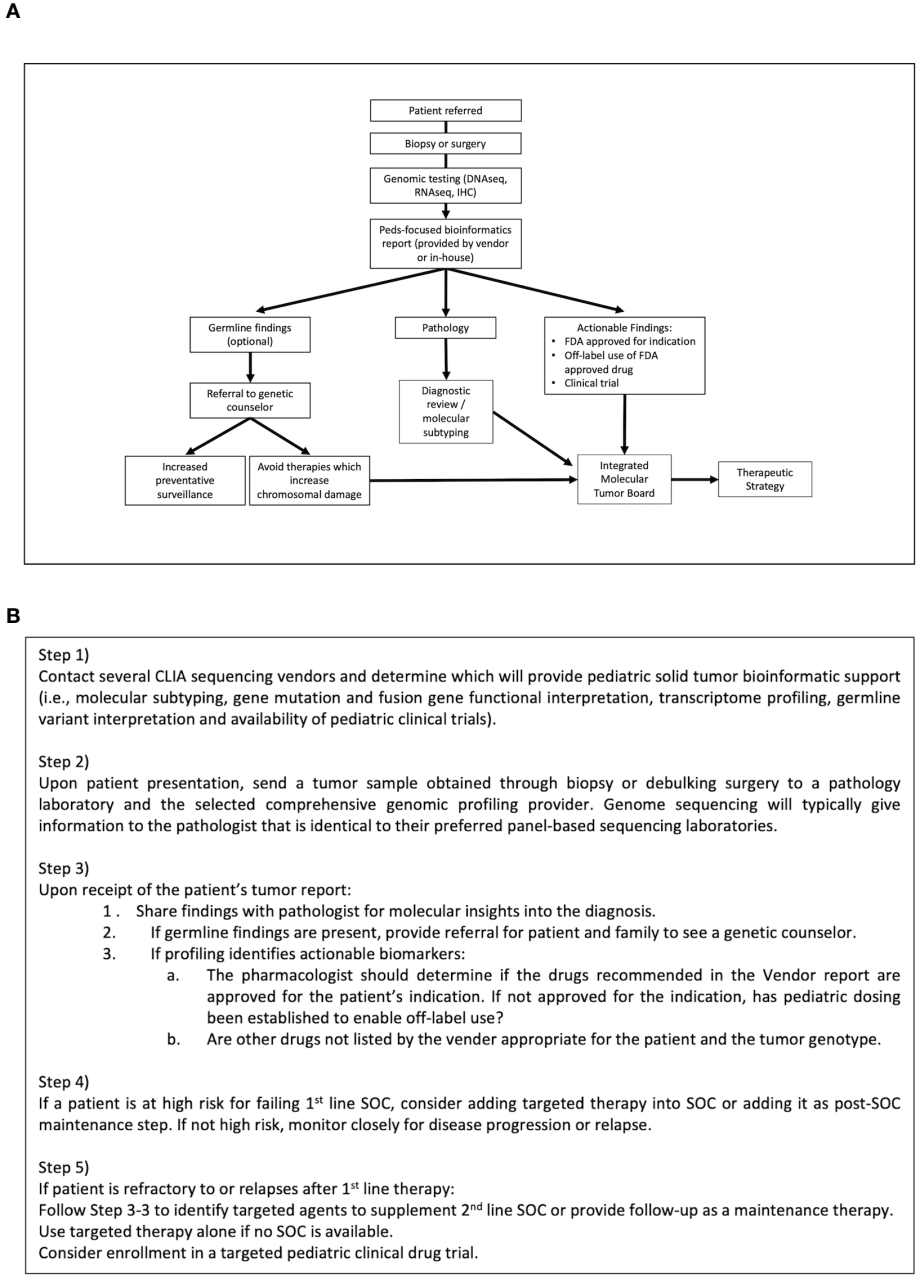
Flow scheme for integrating Precision Oncology into patient care. **(A)** The current precision genomics program protocol at our institution has been reduced to specific steps that can be instituted at any oncology practice in partnership with a skilled pathologist and genetic counselor. **(B)** Stepped guidance for integrating Precision Oncology into existing patient care according to this protocol.

**Figure 2** illustrates our internal flow scheme for adding precision genomics to an existing solid tumor treatment program. Our program has been very successful and can be applied at any level of oncology practice that works closely with a pathologist. We have found that the key steps for success are: 1) selection of a CLIA-certified tumor sequencing laboratory that will provide a comprehensive pediatric-focused genomics report for the ordering oncologist such as Caris, Foundation or Tempus. A good sequencing option for children with newly diagnosed CNS tumors, soft tissue sarcomas or rare tumors is the NCI/COG-sponsored Molecular Characterization Initiative (MCI). MCI provides clinical exome sequencing of paired tumor/normal; DNA methylation array for CNS tumor classification; and targeted RNA sequencing for fusion detection (27). MCI testing is expanding to include soft tissue sarcomas and a subset of other rare cancers. 2) Collaborating with a pathologist who will prepare and provide the best samples for analysis as well as be able to use genomic reports to facilitate the best possible patient diagnosis. 3) Inclusion of a cancer molecular biologist with the ability to interpret genomic reports and to use appropriate online precision oncology databases, such as OncoKB, JAX CKB and ClinicalTrials.gov to identify disease/genome-matched drugs and clinical trials. 4) Partnering with a pharmacist who can recommend pediatric dosing and formulation for targeted drug choices along with suitable drug combinations or maintenance therapy. A pharmacist or a prior authorization specialist is also key in obtaining insurance approvals for off-label drug uses. 5) A strong molecular tumor board willing to integrate precision oncology into the discussion.

## Discussion

Advances in the treatment of pediatric cancer over the past two decades have been inspirational. Five-year survival rates have risen from 58% in the 1970’s to over 80% today (28). This statistic largely reflects improvements in the treatment of hematopoietic cancers while many solid tumor patients have not experienced a parallel survival benefit. Advances notwithstanding, far too many children continue to die from cancer and those who live, often owe their survival to harsh cytotoxic therapies that carry long-term adverse effects (29). With increasing availability and advancements in sequencing technology and clinically active targeted drugs, precision oncology can offer prolongation of life and unexpected cures. While not every patient will benefit from a precision oncology approach, those who do benefit are becoming easier to identify, justifying the time and expense of DNA and RNA sequencing. The experiences of programs like the Precision Genomics Program at Riley Hospital for Children, demonstrate that precision oncology is no longer just an experimental concept, but a viable tool available immediately to help a significant percentage of high-risk pediatric cancer patients. With appropriate clinical and molecular expertise and strong commercial partnerships, pediatric precision oncology can be successfully practiced at small sized institutions.

## Data availability statement

The original contributions presented in the study are included in the article/supplementary material. Further inquiries can be directed to the corresponding author.

## Author contributions

MM: Conceptualization, Data curation, Formal Analysis, Investigation, Methodology, Visualization, Writing – original draft, Writing – review & editing. JI: Investigation, Resources, Writing – review & editing. MS: Conceptualization, Investigation, Supervision, Writing – review & editing. AH: Conceptualization, Investigation, Resources, Writing – review & editing. LL: Data curation, Supervision, Writing – review & editing. JC: Project administration, Writing – review & editing. MF: Conceptualization, Funding acquisition, Supervision, Writing – original draft.
